# Identifiers for the 21st century: How to design, provision, and reuse persistent identifiers to maximize utility and impact of life science data

**DOI:** 10.1371/journal.pbio.2001414

**Published:** 2017-06-29

**Authors:** Julie A. McMurry, Nick Juty, Niklas Blomberg, Tony Burdett, Tom Conlin, Nathalie Conte, Mélanie Courtot, John Deck, Michel Dumontier, Donal K. Fellows, Alejandra Gonzalez-Beltran, Philipp Gormanns, Jeffrey Grethe, Janna Hastings, Jean-Karim Hériché, Henning Hermjakob, Jon C. Ison, Rafael C. Jimenez, Simon Jupp, John Kunze, Camille Laibe, Nicolas Le Novère, James Malone, Maria Jesus Martin, Johanna R. McEntyre, Chris Morris, Juha Muilu, Wolfgang Müller, Philippe Rocca-Serra, Susanna-Assunta Sansone, Murat Sariyar, Jacky L. Snoep, Stian Soiland-Reyes, Natalie J. Stanford, Neil Swainston, Nicole Washington, Alan R. Williams, Sarala M. Wimalaratne, Lilly M. Winfree, Katherine Wolstencroft, Carole Goble, Christopher J. Mungall, Melissa A. Haendel, Helen Parkinson

**Affiliations:** 1 Department of Medical Informatics and Epidemiology and OHSU Library, Oregon Health & Science University, Portland, Oregon, United States of America; 2 European Bioinformatics Institute, European Molecular Biology Laboratory, Wellcome Genome Campus, Hinxton, Cambridge, United Kingdom; 3 ELIXIR Hub, Wellcome Genome Campus, Hinxton, Cambridge, United Kingdom; 4 Berkeley Natural History Museums, University of California at Berkeley, Berkely, California, United States of America; 5 Institute of Data Science, Maastricht University, Maastricht, the Netherlands; 6 School of Computer Science, The University of Manchester, Manchester, United Kingdom; 7 Oxford e-Research Centre, University of Oxford, Oxford, United Kingdom; 8 Institute of Experimental Genetics, Helmholtz Centre Munich, German Research Center for Environmental Health, Neuherberg, Germany; 9 Center for Research in Biological Systems, University of California San Diego, La Jolla, California, United States of America; 10 Babraham Institute, Cambridge, United Kingdom; 11 European Molecular Biology Laboratory, Heidelberg, Germany; 12 Center for Biological Sequence Analysis, Department of Systems Biology, Technical University of Denmark, Lyngby, Denmark; 13 California Digital Library, Oakland, California, United States of America; 14 Science and Technology Facilities Council, Daresbury Laboratory, Warrington, United Kingdom; 15 Genomics Coordination Center, Department of Genetics, University Medical Center Groningen and Groningen Bioinformatics Center, University of Groningen, Groningen, the Netherlands; 16 Scientific Databases and Visualization at Heidelberg Institute for Theoretical Studies, Heidelberg, Germany; 17 Institute for Medical Informatics, Bern University of Applied Sciences, Engineering and Information Technology, Bern, Switzerland; 18 Manchester Institute of Biology, University of Manchester, Manchester, United Kingdom; 19 Department of Biochemistry, Stellenbosch University, Stellenbosch, South Africa; 20 Manchester Centre for Synthetic Biology of Fine and Speciality Chemicals, University of Manchester, Manchester, United Kingdom; 21 Environmental Genomics and Systems Biology, Lawrence Berkeley National Laboratory, Berkeley, California, United States of America; 22 Leiden Institute of Advanced Computer Science, Leiden University, Leiden, the Netherlands

## Abstract

In many disciplines, data are highly decentralized across thousands of online databases (repositories, registries, and knowledgebases). Wringing value from such databases depends on the discipline of data science and on the humble bricks and mortar that make integration possible; identifiers are a core component of this integration infrastructure. Drawing on our experience and on work by other groups, we outline 10 lessons we have learned about the identifier qualities and best practices that facilitate large-scale data integration. Specifically, we propose actions that identifier practitioners (database providers) should take in the design, provision and reuse of identifiers. We also outline the important considerations for those referencing identifiers in various circumstances, including by authors and data generators. While the importance and relevance of each lesson will vary by context, there is a need for increased awareness about how to avoid and manage common identifier problems, especially those related to persistence and web-accessibility/resolvability. We focus strongly on web-based identifiers in the life sciences; however, the principles are broadly relevant to other disciplines.

## Introduction

**Fig 1:**
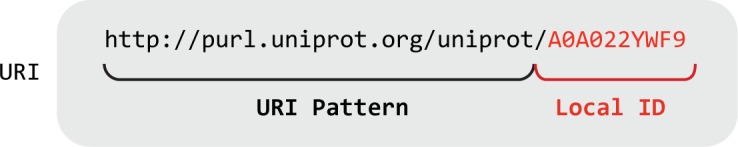
Anatomy of a web-based identifier. An example of an exemplary unique resource identifier (URI) is below; it is comprised of American Standard Code for Information Interchange (ASCII) characters and follows a pattern that starts with a fixed set of characters (URI pattern). That URI pattern is followed by a local identifier (local ID)—an identifier which, by itself, is only guaranteed to be locally unique within the database or source. A local ID is sometimes referred to as an “accession.” Note this figure illustrates the simplest representation; nuances regarding versioning are covered in Lesson 6 and Fig 5.

The issue is as old as scholarship itself: readers have always required persistent identifiers in order to efficiently and reliably retrieve cited works. “Desultory citation practices” have been thwarting scholarship for millennia [1] whether because reliable identifiers were unavailable or because authors failed to use them. While the internet has revolutionized the efficiency of retrieving sources, the same cannot be said for reliability: it is well established that a significant percentage of cited web addresses go "dead" [2]. This process is commonly referred to as link rot because availability of cited works decays with time [3,4]. Although link rot threatens to erode the utility and reproducibility of scholarship [5], it is not inevitable: link persistence has been the recognized solution since the dawn of the internet [6]. However, this problem, as we will discuss, is not at all limited to referencing journal articles. The life sciences have changed a lot over the past decade as the data have evolved to be ever larger, more distributed, more interdependent, and more natively web-based. This transformation has fundamentally altered what it even means to “reference” a resource; it has diversified both the actors doing the referencing and the entities being referenced. Moreover, the challenges are compounded by a lack of shared terminology about what an “identifier” even is. Fig 1 delineates the key components of an identifier used throughout this paper; all technical terms are in fixed-width font and defined in the glossary (S1 Table).

An identifier is a sequence of characters that identifies an entity. The term “persistent identifier” is usually used in the context of digital objects that are accessible over the Internet. Typically, such an identifier is not only persistent but also actionable [7]: it is a Uniform Resource Identifier (URI)[8], of type hypertext transfer protocols (http/s), that, at a minimum, you can paste in a web browser address bar and be taken to the identified source. Formally breaking down a URI into these two components (URI pattern and local identifier [local ID], as shown Fig 1) makes it possible for meta resolvers to “resolve” entities to their source. This practice also facilitates the representation of a URI as a compact URI (CURIE), an identifier comprised of <Prefix>:<Local ID> wherein prefix is deterministically convertible to a URI pattern and vice-versa. For instance, the above URI could be represented as uniprot:A0A022YWF9. This deterministic conversion makes it easy for meta resolvers as well, (e.g., http://identifiers.org/uniprot:A0A022YWF9).

Suboptimal identifier practice is artificially constraining what can and cannot be done with the underlying data: it not only hampers findability, accessibility, interoperability, and reuse (FAIR principles) [9,10], but also compromises mechanisms for credit and attribution. This article seeks to provide pragmatic guidance and examples for how actors in life science research should handle identifiers. Optimizing web-based persistent identifiers is harder than it appears. There are a number of approaches that may be used for this purpose, but no single one is perfect. Identifiers are reused in different ways for different reasons, by different consumers. Moreover, digital entities (e.g., files, such as an article), physical entities (e.g., tissue specimens), living entities (e.g., Dolly the sheep), and descriptive entities (e.g., “mitosis”) have different requirements for identifiers [11].

**Fig 2:**
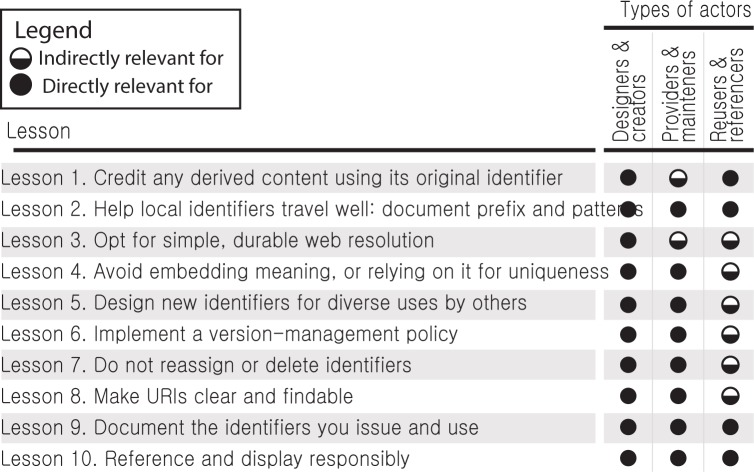
A summary of the 10 recommendations and their direct or indirect impact on different kinds of identifier roles.

The problem of identifier management is hardly unique to the life sciences; it afflicts every discipline from astronomy [3] to law [12]. Towards this end, several groups (S1 Text) have been converging on identifier standards that are broadly applicable [9,13–15]. Building on these efforts and drawing on our experience in integrating and accessing data from a large number of sources, we outline the identifier qualities and the best practices that we consider to be particularly important in the context of large-scale data integration in the life sciences. In Lessons 1 through 9 (Fig 2), we propose actions for data providers when designing new identifiers, maintaining existing identifiers, as well as when reusing and referencing identifiers from other datasets. In Lesson 10, we conclude with guidance for data integrators and redistributors on how best to reference multiple identifiers from diverse sources. More often than not, life science data providers often invent or organically grow their own identifier systems without a firm grasp of the lasting implications. Data providers are urged to take a long-term view of the scope and lifecycle of data and the identifiers that they issue, and to consider using existing identifier platforms and services [14] where appropriate.

Throughout this document, the word “must” is reserved for practices that ensure against the collision, ambiguity, or inaccessibility of items referenced by identifiers; instances of “must” are also often specific to particular design choices. We use the word “should” to convey that the trade-offs must be understood and carefully weighed before choosing a different course (e.g., consistent with IETF RFC2119 [16]).

There is no one in science that is unaffected by identifiers. Fig 2 details 3 basic roles one might play in the scholarly landscape and how identifiers are relevant in these contexts. Who are designers and creators? These are databases, but also those that submit supplemental data to archives, and anyone creating structured data. Who are the providers and maintainers? These are databases as well, but also services and indices that support web resolution and data validation. Who are the reusers and referencers? These are the “research data parasites” [17], but also your average author: while authors may specify an identifier for a resource (e.g., a gene or antibody), more often identifiers are contextually inferred by the journals or curators, whether pre- or postpublication.

Many of the following recommendations are applicable during the planning and identifier conceptualization phase, i.e., before any identifiers are created. The retrofitting (especially Lessons 1, 4, 5, and 6) of existing identifiers can sometimes be too difficult or may even make matters worse: for instance, changing existing identifiers introduces the need for systems that can recognize the variations for what they are; such overhead can outweigh potential benefits. Each of the lessons is relevant to the basic classes of identifier actions (design, provision, reuse (Fig 2) within the ecosystem of diverse data providers and integrators. Even if we largely agree on what makes for a good persistent identifier (Table 1), actual implementation often falls short. No provider is perfect and no two are alike, hence the objective is to learn from each other’s diverse experiences. All of the negative examples herein are anonymized variations of real-world identifiers that we have had to work with.

**Table 1:** Desirable characteristics for database identifiers in the life sciences.

Characteristics	Definition	General rationale/impact on data integration	Specific example of a possible ramification due to non-adherence
**Unambiguous**	One Local ID must be associated to no more than one entity locally. One URI must be associated to no more than one entity globally.	Avoids collisions that result in integrating on the wrong entity.	A physician uses a wrong clinical guideline and makes a wrongful diagnosis because the info button within the clinical information system is linked to the wrong document.
**Unique**	One entity should ideally be identified by no more than one URI.	(1) Eliminates the cost of maintaining public mappings between equivalent identifiers (2) Avoids false negatives if data integrators do not leverage or know about a mapping.	A researcher fails to make a pathway discovery because she does not realize that http://mydb.org/1234567 and http://mydb.org/q?=1234567 are in fact the same.
**Stable (identifier)**	The URI, and by extension the local ID, should wherever possible stay the same over time.	Avoids link rot.	A researcher is unable to reproduce an experiment because the link to a record is dead.
**Stable (entity)**	Identifier must NOT be reassigned to an altogether different entity, though the original entity may evolve provided a change history is documented.	Avoids integrating on the wrong entity.	Because a new chemical gets an old ID, a chemist uses the wrong chemical in a reaction.
**Version- documented**	If the entity’s definition or essential metadata changes substantially, (Lesson 7) the identifier should, wherever possible be versioned and/or change history documented.	Avoids integrating on the wrong entity state (specified through version).	A given experiment is not reproducible because the specific build version of a gene sequence was not specified.
**Persistent**	The identifier must NOT be deleted (but may be deprecated).	Avoids link rot.	Information about a gene model is completely lost.
**Web-resolvable**	The URI must be resolvable to a web address where the data or information about the entry can be accessed.	Avoids the unnecessary proliferation of resolvable identifiers issued by third parties (for entities that are not resolvable and/or not identified in their native context) See also surrogate identifier.	A dozen different third-party providers mint identifiers for entities that are not actually under their control. Harmonization between these off-brand identifiers is painful.
**Convertible**	The local ID and its URI counterpart must be inter-convertible by applying the URI pattern to the local ID. Note that in some communities (e.g., ontologies), the local ID is often a CURIE by default.	Avoids the need for special handling of edge cases when integrating data at scale.	Data integrators spend time cleaning identifiers and handling edge-cases instead of doing science.
**Defined**	The total set of assignable identifiers for the database must be describable through a formal pattern (regular expression).	Facilitates validation and extraction from scientific text, thus the pattern should be as tightly specified as possible (see Lesson 3).	Identifiers cannot be validated and a provider may find it hard to assess their impact in the literature.
**Web-friendly**	The local ID should wherever possible be of a format that does not need special handling when used in URL and common exchange formats (e.g., XML).	Avoids potential points of failure due to malformed URL, XML, etc.	Use of the identifier produces malformed XML and/or requires special detection and encoding.
**Free to assign**	The identifier should ideally be assigned at no cost to individuals depositing data in a repository.	Lowers barriers for data generators to deposit data.	Data generators become reluctant to deposit data in order to minimize costs.
**Open access and use**	The identifier and its label should be able to be transparently referenced and actioned (e.g., in a public index or search) anywhere by anyone and for any reason. Restrictions on associated data may apply but are not recommended.	Enables integration on the basis of scientific merit, rather than on the restrictions of the license.	When there are license restrictions on the identifier and/or label (not just the content) it thwarts meaningful reuse and redistribution of whole datasets.
**Documented**	The identifier scheme should be documented.	Encourages consistent use of existing identifiers by others and reduces the number of ways identifiers are represented.	Inconsistent informal approaches to referencing are difficult to harmonize post-hoc. By extension, impact is harder to assess.

CURIE, compact uniform resource identifier; Local ID, local identifier; URI, unique resource identifier; URL, uniform resource locator; XML, extensible markup language.

### Lesson 1. Credit any derived content using its original identifier

**Fig 3:**
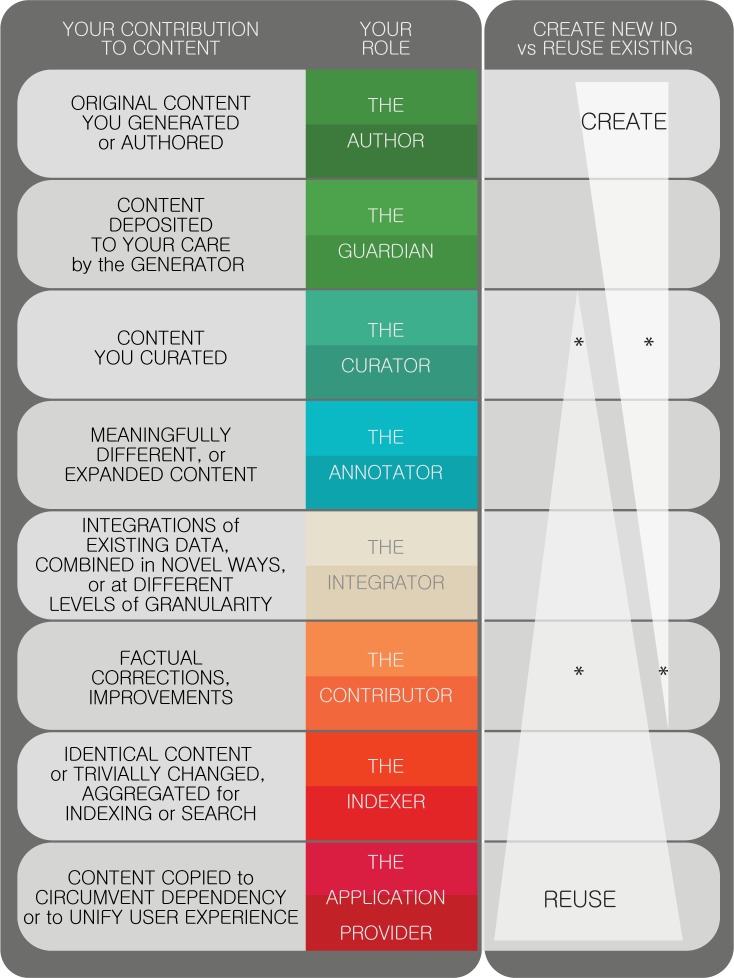
Contributions and roles related to content as they correspond to identifier creation versus identifier reuse. The decision about whether to create a new identifier or reuse an existing one depends on the role you play in the creation, editing, and republishing of content; for certain roles (and when several roles apply) that decision is a judgement call. Asterisks convey cases in which the best course of action is often to correct/improve the original record in collaboration with the original source; the guidance about identifier creation versus reuse is meant to apply only when such collaboration is not practicable (and an alternate record is created). It is common that a given actor may have multiple roles along this spectrum; for instance, a given record in monarchinitiative.org may reflect a combination of (a) corrections Monarch staff made in collaboration with the original data source, (b) post-ingest curation by Monarch staff, (c) expanded content integrated from multiple sources.

If you manage an online database (repository, registry, or knowledgebase), consider its role in identifying and referencing the knowledge that it publishes. We advise that you only create your own identifiers for new knowledge (Fig 3). Wherever you are referring to existing knowledge, do so by using existing identifiers (Lesson 10); otherwise, wherever the 1 to 1 relationship of identifier to entity breaks down, costly mapping problems arise. Whether or not you create a new identifier, it is vital to credit any derived content in a way that includes its indigenous identifiers [11]; to facilitate data integration, all such identifiers should be machine processable and transparently mapped.

### Lesson 2. Help local IDs travel well: Document prefix and patterns

If you reference others’ data or anticipate your data being referenced by others, consider how you document your identifiers. Note that you may not know a priori how your data may be used. Data do not thrive in silos: they are most useful when reused, broken into parts, and integrated with other data, for instance in database cross references (“db xrefs”). In spite of how important identifiers are to this process, the confusion with identifiers often starts with the basics, including what the “identifier” even is. A local ID (Fig 1) is an identifier guaranteed only to be unique in a given local context (e.g., a single provider, a single collection, etc.), and sometimes only within a specific version; as such, it is poorly suited to facilitate data integration because it can collide when considered in a more global landscape of many such identifiers. For instance, the local ID “9606” corresponds to numerous entities whose local accessions are based on simple digits, including a Pubmed article, a CGNC gene, a PubChem chemical, as well as an NCBI taxon, a BOLD taxon, and a GRIN taxon. Local IDs therefore need to be contextualized in order to be understood and accessed (resolved) on the web. This is often accomplished through the use of a prefix, which should be documented. If this is overwhelming, don’t forget that there are meta resolvers and services built to help for exactly this reason (see Lesson 3).

URIs are identifiers that resolve on the web. “Cool URIs don’t change” [6] because when they do change (or disappear) all existing references break. In the context of academia alone, “reference rot” impacts 1 in 5 publications [4]. Despite vulnerability to link rot, the global http/s URI (Fig 1) is the best available identifier form for machine-driven global data integration because (a) the http URI is a widely adopted Internet Engineering Task Force (IETF) standard and (b) the http URI’s uniqueness is ensured by a single well-established name–granting process (DNS). However, the length of URIs can make them unwieldy for tasks involving human readability even within structured machine-parsable documents. CURIEs [18] (Fig 1) are a mature world wide web consortium (W3C) standard that is well established in some contexts (e.g., JSON-LD and RDFa) as they enable URIs to be understood and conveniently expressed. We, the authors, are not absolutist about anyone using CURIEs; however, we agree that the features that make for good URIs also happen to make CURIEs possible (for those who wish to use them; S2 Text).

Thus, if you are a database provider, it is in your best interests to document and preferably register (a) the prefix (Fig 1) that you would like others to use and (b) its binding to a URI pattern (Fig 1). Your chosen prefix should be unique, at least among datasets that are likely to be used in the same context; choose the registry/registries that is appropriate for your data type and discipline; a list of such registries is available in S2 Table together with their corresponding registration uniform resource locators (URLs). PrefixCommons [19] is a platform designed to (a) aggregate prefix mappings from primary registries to enable these registries to make more informed decisions about which new prefixes to issue and (b) for any given integrator to publish the set of mappings that they happen to use. In the context the life sciences, Identifiers.org [20] is the most important location to register a prefix for digital/data objects not already resolved by doi.org; similarly OBOfoundry [21] and Bioportal [22] are the most important for ontology prefixes. These authorities guarantee prefix uniqueness within their respective remit, are beginning to better coordinate, and are setting the standards for how prefixed identifiers are referenced in the literature.

### Lesson 3. Opt for simple, durable web resolution

**Fig 4:**
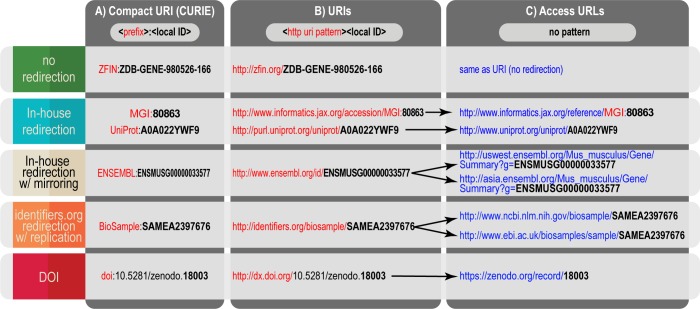
Examples of provisioning resolvable Unique Resource Identifiers (URIs). Compact URIs (CURIEs; Panel A), URIs (Panel B), and Access URLs (Panel C) with no redirection (the Zebrafish Identification Network [ZFIN]), in house redirection (UniProt and Ensembl), and third party resolvers (using identifiers.org and digital object identifiers [DOI]). In each case, the URI can be algorithmically derived from the CURIE because the local identifier (local ID) portion itself is included (unmodified) within the URI. Access URL design patterns differ substantially by provider and may change over time. As long as access URLs (and other ephemeral links) are not used as the referenced identifier, they can include prefix and colon (Mouse Genome Informatics [MGI]) or not (Ensembl), they may include the entire local ID (Biosample) or not (DOI), and they may include type (MGI) or not (ZFIN).

A core component of persistent identification is redirection, the absence of which makes it extremely difficult to provide stable identifiers. When designing (or refining) your http URI strategy:

- Consider a resolution provider before doing it yourself. If you are a database provider, you must implement an http URI pattern (Fig 3B) for local IDs to be resolvable to a web page. If you choose to outsource to a resolver service, use an approach that adheres to best practice [14] (e.g., digital object identifier ([DOI] DataCite, CrossRef), Identifiers.org, Handle.net, PURL (now via InternetArchive), EPIC, ARK) and be mindful of your constraints regarding cost, metadata ownership, turnaround time, versioning support, etc (see S3 Text for a more comprehensive list of considerations). Some of these resolver services can even provide content negotiation for different encodings of your data [14] and make it easier to provide direct access to data, metadata, and persistence statements [23]. If you have the resources to support your own persistent URIs, design these to be “cool” [6]; this is most easily achieved by keeping URIs simple.
- Avoid inclusion of anything that is likely to change or lapse, including administrative details (e.g., grant name) or implementation details such as file extensions (“resource.html”), query strings (“param = value”), and technology choices (“.php”). Never embed the local ID in the query part of a URI e.g., http://example.com/explore?record=A123456.
- Omit trailing characters after the local ID. In all cases, the URI pattern must include the protocol (e.g., https://) and, if applicable, trailing slash or other delimiters. Trailing characters after the local ID are strongly discouraged as they unnecessarily increase the variability with which the identifier is represented and also complicate straightforward appending of the local ID (requiring that tokens such as $id hold the place of the local ID in the URI pattern e.g., http://example.com/$id/view.do).
- Avoid unnecessary detail. Detail in “persistent” identifiers creates complexity that must be managed in perpetuity. Make every attempt to limit the degree of path nestedness (e.g., do http://example.com/A123456 rather than http://example.com/vertebrates/mammals/rodents/rat/white-rat/A123456); see also Lesson 5 regarding types and meaning. The CURIE approach can work with any resolver(s): see for instance examples 4 and 5 in Fig 4. By choosing a single URI pattern, you make it possible for others to resolve your identifiers simply (Fig 4A) without their having to know the type and its syntax in http URI. See also Lesson 4 regarding omission of semantics.

Despite their differences, the examples in Fig 4 share the most important features above.

### Lesson 4. Avoid embedding meaning or relying on it for uniqueness

When designing new local IDs or http URIs, avoid embedding meaning or relying on it for identifier uniqueness. Instead, favor opaque identifiers and convey meaning in the entity’s metadata; some metadata (such as resource type) can and should be conveyed in the HTTP header where possible [24]. The structure and scope of collections evolve, as does scientific understanding; minimizing the meaning embedded in identifiers makes them less vulnerable to obsoletion. In human genetics, many genes were initially identified based on disease association; later the identification, nomenclature, and function of genes were separated into different activities. Meaning should only be embedded if it is indisputable, unchangeable and also useful to the data consumer (e.g., computer-processable). For instance, the type of entity imparts meaning to users and may fulfil these 3 criteria. When encountered, typing may be embedded, either within the local ID (ENSMUSG…), or within the http URI path (…/gene/12345), or both. In any case, if you opt to include type in the identifiers you issue, avoid relying on type for uniqueness: that is to say, once a local ID (e.g., 12345) is assigned it should never be recycled for another entity, even an entity of a different type (e.g., …/gene/12345 and …/patient/12345).

If you need the ability to convey meaning in a dense character space, you don’t need to do so in the identifier itself; consider instead implementing an entity label, for instance as is done in model organism nomenclature such as by Mouse Genome Informatics (MGI; label: Kit^W^/Kit^W-v^, id: MGI:2171276). Labels are for human readability only; even if they are deemed durable, labels should not be treated as identifiers, nor should they appear within http URIs. URI patterns, if type-specific, require a corresponding type-specific prefix. For example, the Library of Integrated Network-based Cellular Signatures (LINCS) contains entities of several types including cells and proteins. Cell records are resolved using the pattern http://lincs.hms.harvard.edu/db/cells/, whereas protein records are resolved using the pattern http://lincs.hms.harvard.edu/db/proteins/; thus, if more than one lincs type is referenced/integrated in the same context, it requires the use of two different prefixes, e.g., such that lincs.cells:50001 → http://lincs.hms.harvard.edu/db/cells/50001 and lincs.protein:200001 → http://lincs.hms.harvard.edu/db/proteins/200001, respectively. By contrast, MGI implements a single prefix for all types of entities in their corpus (genes, markers, alleles, etc); accordingly, this prefix “MGI” corresponds to a single URI pattern (http://www.informatics.jax.org/accession/). Thus the single MGI resolver works for all accessions, regardless of type, and redirects them to their corresponding type-specific destination (e.g., MGI:2442292 → http://www.informatics.jax.org/accession/MGI:2442292 which redirects to http://www.informatics.jax.org/marker/MGI:2442292) all without the user needing to know the type beforehand. Dual approaches like MGI’s can be helpful to different kinds of consumers: type-agnostic resolution is useful in cases such as data citation in the literature where (a) the type of the identified entity is not of primary importance, or (b) the type of the entity is already conveyed contextually, and/or (c) where resolution is done systematically at scale and/or involves many and varied or volunteer contributors that may be difficult to coordinate. Type-specific resolution is useful in cases like bioinformatic research pipelines where embedded type may facilitate the human-led debugging process. If you support both kinds of resolution, it is best to document (a) whether you intend for both to be treated as persistent and (b) what mapping support you provide. Note that while type-agnostic resolution has important advantages, it must only be undertaken if all local IDs of any type, past and future, can be guaranteed to never collide.

Whether or not your URIs or your local IDs include type, you should provide other ways for humans and machines to determine the type of entity that is being identified; this is most often achieved via web services (e.g., as done in the Monarch Initiative), but ideally also within metadata landing pages [23,25], if provided.

### Lesson 5. Design new identifiers for diverse uses by others

Preexisting identifiers should be referenced without modifications (see Lesson 10). However, if you create new local IDs, there are some design decisions that can facilitate their use in diverse contexts (spreadsheets, other databases, web applications, publications, etc.).

- Avoid problematic characters. Local IDs should, wherever possible, comprise only letters, numbers, and URL-safe delimiters. Omission of other special characters guards against corruption and mistranscription in many contexts; however, it is acceptable that the local ID be in CURIE format because modern browsers resolve colons without having to encode them. Although characters “/” and “?” are technically URL-safe, they are very problematic when used within the local ID, as these characters are assumed to have special meaning and can complicate parsing of the identifiers, whatever forms they take. For the same reason, local IDs should ideally not contain the dot character (“.”) except to denote version where appropriate (see Lesson 7).
- Define a formal pattern and stick to it. Local IDs must adhere to a formal pattern (regular expression); this facilitates the validation of URIs and improves the accuracy of mining identifiers from scientific text. Consider a fixed length of 8–16 characters (according to the anticipated number of required local IDs). A pattern may be extended if all available identifiers are issued, but existing identifiers should not be changed. To minimize local ID collisions at a global scale, it is considerate to tightly specify your pattern (e.g., by using one or more fixed letters). The regular expression should include a fixed, documented case convention. In most cases, it is advised that identifiers not rely on case for their uniqueness: if you assign ab-12345 to one entity and AB-12345 to a different entity, collisions due to mistranscription are more likely. Case-sensitive patterns are best reserved for when brevity is a constraint, and hand transcription is not (e.g., millions of IDs are required and each ID has to be short enough to be printed on a vial label).
- Avoid problematic patterns. Consider using both letters and numbers in the local ID, but if you do use both consider omitting characters that can be mistranscribed [26] This avoids misinterpretation as numeric data (e.g., the truncation of leading zeros or conversion to exponents in spreadsheets). Some patterns can result in misinterpretation and/or corruption whether as dates (e.g., “may-15”), exponents (e.g., “5e1234”) [27], or as unintended words (e.g., “bad-12”). Such issues in gene names alone have been shown to impact 19% of life sciences papers [28]. A historically common, if thorny, identifier pattern is that of “_” and “:” are often interconverted and it has come to be understood as compact notation, delimiting the prefix from the rest of the identifier. Therefore “_” or “:” should (a) occur no more than once per identifier, and (b) should only be used if local IDs are intended to be deterministically expanded to a resolvable http URI. For instance, if your intended prefix is “MyDB”, then either MyDB:gene-6622 or MyDB_gene-6622 are acceptable patterns, but MyDB_gene_6622 is problematic, as it could result in three possible conversions by others, even if these are not intended: MyDB_gene:6622, MyDB:gene_6622, MyDB:gene:6622. Whatever pattern you adopt, document which variations you support resolution of, if any.

### Lesson 6. Implement a version-management policy

Whether you produce original data, or reference data from others, consider the impact of data changes. The nature, extent, and speed of data changes impact how data can be referenced and used. Document your chosen version management practice: if you issue identifiers, the change history for the entity should be either documented or retrievable with a URL-based query. Alternatively, the identifier itself can be versioned whether or not change history is also supported.

Embedding versioning in identifiers is recommended if the prevailing use of an unversioned identifier results in “breaking changes” (e.g., a change in the hypothesized cause of a disease). However, if new information about the entity emerges slowly and the changes are “nonbreaking”, it is reasonable to instead maintain a machine-actionable change history in the entity’s metadata. The history should be a list of changes categorized in such a way that users can assess the impact on their work. Versioning and change history work well together, especially when multiple types of changes overlap. Even where previous records are entirely removed, the URI should continue to resolve, but to a “tombstone” page (Lesson 7). A resource should communicate clearly what a version change refers to. UniProt and RefSeq use versions to reflect changes in sequence. Ensembl uses versions to reflect changes in sequence and splicing for transcript records but sequence alone for protein records. In each of these examples, the changes in the annotations that are attached to a record does not alter the version.

**Fig 5:**
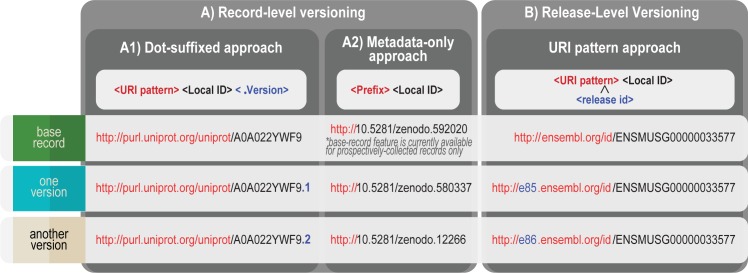
Record-level versioning and release-level versioning.

There are two approaches to versioning: record-level (Fig 5A) and release-level (Fig 5B); the latter is more common in the life sciences. Release-level versioning is usually performed for defined data releases. However, use cases vary; some user communities need to resolve individual archived entities via a deterministically-versioned URI pattern, for example as is done in Ensembl (e.g., http://e85.ensembl.org/id/ENSMUSG00000033577). If you do not have the ability (or common use case) to maintain individually resolvable-archived records, we strongly recommend that you (a) support export to files so that users can archive the records they need, and (b) make snapshots available for the database, whether in whole or in parts [29].

If you version identifiers at the level of the individual record, the most common approach in the life sciences is to version in the local ID after the “dot”, as per UniProt in Fig 5A1 and Table 2. Maintaining version information solely in metadata (e.g., without suffixing) is possible; this approach is truer to Lesson 4 (“Avoid embedding meaning”) but is also so technically difficult that few providers do it well. To our knowledge thus far, Zenodo.org is the only provider that comprehensively supports the metadata-only versioning (shown in Fig 5A2); moreover, they introduced this feature in 2017, four years after their launch and, for prospectively-collected records only. In metadata-only versioning, a completely new Local ID is used for each new version; ideally there is also a single base identifier to which each version is directly linked. While there is yet no standard for how version metadata should be structured, there must be some mechanism for machines to obtain the identifier that corresponds to the most recent version of the record. We strongly recommend providing a transparent and machine-readable mapping between identifiers, together with a deterministic mechanism for machines to obtain the latest version of the record (e.g., via respresentational state transfer [REST] application programming interface [API] or by inserting “/latest/” in the URI path). Although the topic of when and how to version data is of great interest, use cases vary and consensus is elusive. Other groups have discussed change management consideration and “content drift” in more depth [2,30,31].

**Table 2:** Recommendation for versioning.

	Recommendation	UniProt	RefSeq	Ensembl
General versioning practices	Primary versioning strategy	Record level	Record level	Release level
	Past versions are accessible	All versions of individual records are accessible http://www.uniprot.org/uniprot/P12345?version=* http://www.ebi.ac.uk/uniprot/unisave/app/#/	All versions of individual records are accessible https://www.ncbi.nlm.nih.gov/nuccore/NM_004333.4?report=girevhist	Maintains all archives for at least 5 years; some key releases may be maintained for longer. All databases maintained for at least 10 years (currently all databases available from 2004) http://www.ensembl.org/info/website/archives/index.html
	Release versioning available	ftp.ebi.ac.uk/pub/databases/uniprot/previous_releases	No past releases available	ftp.ensembl.org/pub and archive sites
	Documentation exists regarding what kinds of record changes prompt a new version to be issued.	http://www.uniprot.org/help/entry_history http://www.uniprot.org/help/uniprotkb http://www.uniprot.org/help/fasta-headers	https://www.ncbi.nlm.nih.gov/books/NBK50679/#RefSeqFAQ.what_causes_the_version_number	http://www.ensembl.org/info/genome/stable_ids/index.html
URL version practices	The base identifier (the one with no explicit version) should resolve (302 redirect) to most recent version.	http://www.uniprot.org/uniprot/P12345	https://www.ncbi.nlm.nih.gov/nuccore/NM_004333	http://ensembl.org/id/ENSMUSG00000033577
	Base identifier should be deterministically convertible from any other version.	Remove dot suffix from the Local ID, e.g.: http://www.uniprot.org/uniprot/P12345**.1** to http://www.uniprot.org/uniprot/P12345	Remove dot suffix from the Local ID, e.g.: https://www.ncbi.nlm.nih.gov/nuccore/NM_004333.4 to https://www.ncbi.nlm.nih.gov/nuccore/NM_004333	Remove build number from the URI, e.g.: http://e85.ensembl.org/id/ENSMUSG00000033577 to http://ensembl.org/id/ENSMUSG00000033577
	Older versions must resolve.	http://www.uniprot.org/uniprot/P12345.**1**	https://www.ncbi.nlm.nih.gov/nuccore/NM_004333.1	http://e85.ensembl.org/id/ENSMUSG00000033577
	Illegal or invalid version should produce an informative http error code and a HTML page explaining the error.	http://www.uniprot.org/uniprot/P12345.302 returns a 400 bad request and brief description	https://www.ncbi.nlm.nih.gov/nuccore/NM_004333.302 returns a 404 page not found	Error not returned
	A list of all previous versions should be available.	See “history” tab in user interface	See format dropdown in user interface	http://www.ensembl.org/info/website/archives/assembly.html
	Link from older version to current version should ideally be provided.	P12345.3	Link available at the top of the page	Plans to support
	Two versions (or dates) should ideally be comparable.	Record history provides comparison	Record history provides comparison	Unsupported

Local ID, local identifier; URI, unique resource identifier; URL, uniform resource locator.

### Lesson 7. Do not reassign or delete identifiers

Identifiers that you have exposed publicly, whether as http URIs or via APIs, may be deprecated but must never be deleted or reassigned to another record. If you issue identifiers, consider their full life cycle: there is a fundamental difference between identifiers which point to experimental datasets (GenBank/ENA/DDBJ, PRIDE, etc.) and identifiers which point to a current understanding of a biological concept (Ensembl Gene, UniProt record, etc.). While experimental records are less likely to change, concept descriptions may evolve rapidly; even the nature and number of the relevant metadata fields change over time. Moreover, the very notion of identity is often strongly impacted by relationships (e.g., between concepts or processes).

Extensive changes cannot be captured with numerical suffixing alone. For instance, taxonomists may split or merge species, pathologists may split or merge diseases, or hypothesized entities may be proven not to exist (e.g., vaccine-induced autism). Global initiatives (S1 Text) are actively exploring identifier strategies for such use cases. In the meantime, consider Table 3 recommendations.

**Table 3:** Recommendations for identifier lifecycle management.

Recommended Handling	Example
**Obsoletion:** If an entry has been removed or deprecated, the original identifier must still resolve to a “tombstone page”. Reasons for obsolescence should be indicated. If the obsoleted ID is replaced by another ID, the replacement must be present and also described as automatic or suggested, preferably using some controlled vocabulary, for instance the ontology properties iao:replaced_by and obo:consider, respectively. The standards for this are still evolving. The obsoleted ID must never be reassigned to another entity. A list of obsoleted IDs should be maintained.	Single obsoleted identifier: http://www.uniprot.org/uniprot/A0AV18 List of obsoleted identifiers: uniprot.org/help/deleted_accessions
**Merging:** When 2 or more identifiers are merged, a new recipient identifier should be designated as the primary (citable) one and should contain information about the legacy identifiers it encompasses. Any legacy identifiers should continue to resolve via redirection to the primary identifier.	UniProt entries Q57339 and O08022 have been merged into Q00626. Q57339 and O08022 are redirected to Q00626.
**Splitting:** If an identifier is split (demerged) into 2 or more new ones, new identifiers should be assigned to all the new entries. The legacy identifier must be marked as obsolete, but must also still resolve, providing a warning and pointers to the new ones as per above.	UniProt entry P29358 has been split into P68250 and P68251. P29358 displays a warning and links to the demerged entries: http://www.uniprot.org/uniprot/P29358

ID, identifier.

### Lesson 8. Make URIs clear and findable

Persistent URIs almost always differ from the ephemeral URLs to which users are ultimately directed (Fig 4). Therefore, whether you produce original data, or reference others’ data, make persistent URIs obvious to users so that they are less inclined to copy the link that appears in the browser address bar. As a group, the best practitioners of this lesson are currently academic journals; they prominently advertise the DOI corresponding to each article. In situations where the version of a data record matters, advertise the corresponding permanent link (permalink) together with a statement about persistence. E.g.:

“The permanent link to this page, which will not change with the next release of Ensembl is: http://e85.ensembl.org/id/ENSMUSG00000033577 We aim to maintain all archives for at least five years; some key releases may be maintained for longer”

**Fig 6:**
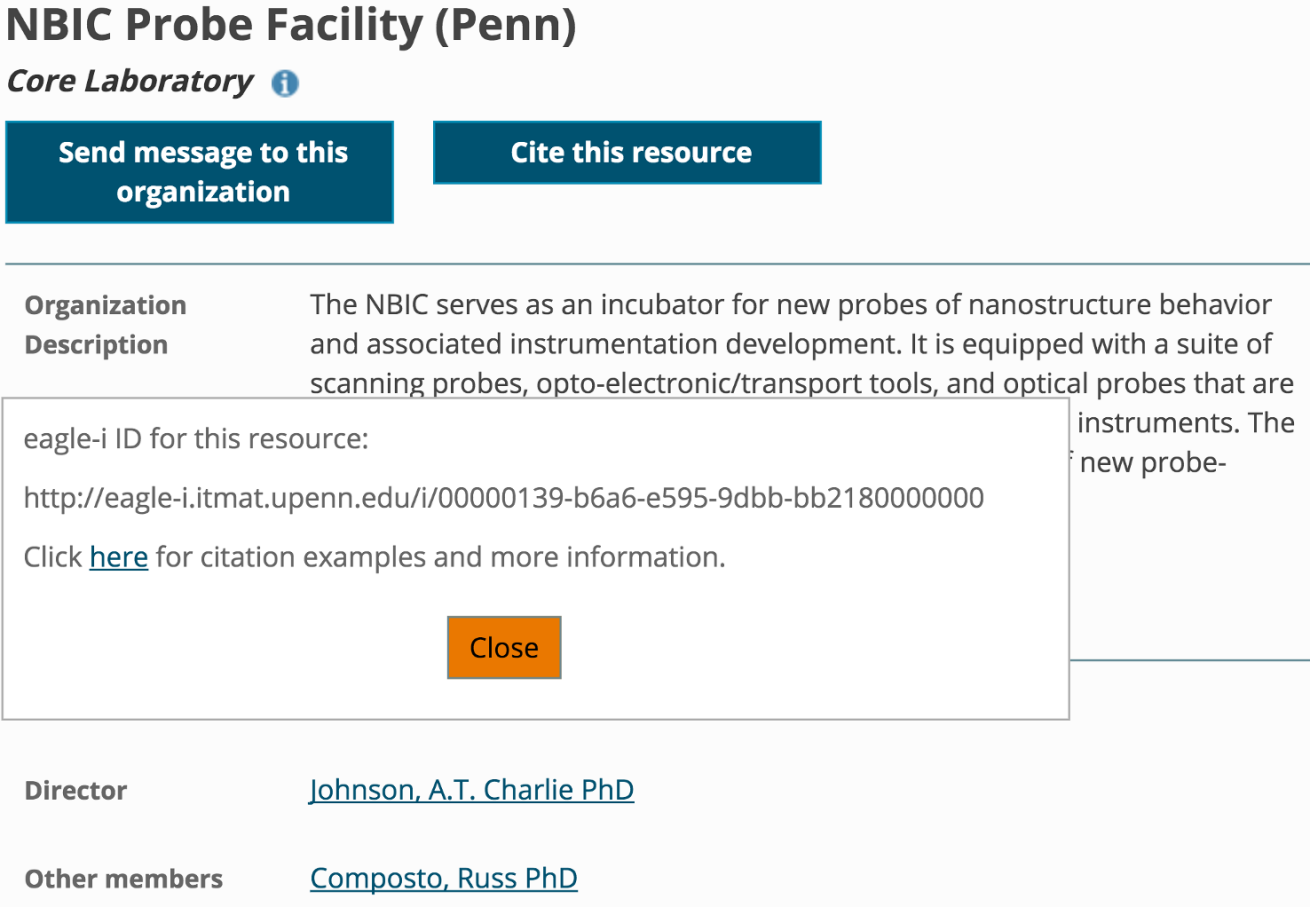
Eagle-i record-level citation widget.

For archived records that are out of date, make this clear to the user and provide a link to the updated version (see http://www.uniprot.org/uniprot/P12345.1, for instance). Although it is good practice for each database website to include general citation guidance for users [32], it is increasingly important to provide a prepopulated citation at the level of each record. When it comes to making record-level citation clear on every page, eagle-i [33] provides the best example of a primary data source that we know of (outside of providers that issue DOIs; Fig 6). Additional features that are useful in such widgets are that full references should be copy-pastable, integrated with reference managers, and pre-populated with the version information and access date.

### Lesson 9. Document the identifiers you issue and use

The global-scale identification cycle is a shared responsibility and provider and/or consumer roles often overlap in the context of data integration. Whether you issue your own identifiers or just reference those of others, you should document your identifier policies. S3 Table provides a set of questions that data providers and redistributors can use to develop such documentation. Documentation should be published alongside and/or included together in a dataset description, for instance, as outlined in the recommendations for Dataset Descriptions developed by the W3C Semantic Web in the Health Care and Life Sciences Interest Group [34]. For examples of such documentation see ChEMBL [35] and Monarch [36]; the format may vary.

### Lesson 10. Reference and display responsibly

The final lesson describes referencing recommendations for data redistributors: data aggregators, who collect information from different sources and redisplay it; data publishers, who disseminate scientific knowledge through publications; and online reference material such as WikiData [37].

When external entities are referenced in narrative online text, they should be hyperlinked where possible, either to their URIs or to a destination that documents their URIs. Access URLs are volatile (see Lesson 4) and must not be used for referencing or linking in any context intended to persist.

Broader issues associated with citation of data and software in the traditional literature are outside of the scope of this paper, but S1 Text lists relevant complementary efforts. Our recommendations regarding data citation in the literature are circumscribed: within static documents of record (e.g., in portable document formats [PDFs]), or in situations where link updates are costly and/or difficult, we strongly advocate always using the URLs of well-established third-party resolvers, whether they be primary resolvers such as doi.org or hdl.net or meta-resolvers such as Identifiers.org, or n2t.net (S2 Table). Each provider has a corresponding URI pattern; however, those URIs can and do change over time. Third-party resolvers are not immune to change; the fact that the PURL.org resolver recently nearly sunset into “read-only” mode illustrates (a) the importance of sustained community buy-in and governance, and (b) that reliance on third parties for resolution is not without its risks. Nevertheless, the risk that URIs will break because of resolver change is modest and easier to mitigate compared to the risk that any single referenced collection will move or disappear. It is incumbent on meta-resolvers to be vigilant about detecting and updating their redirection rules in the face of provider changes. Identifiers.org is able to redirect to one of a few potential provider destinations based on an algorithm that considers (a) provider uptime and (b) whether a given provider is a “primary” source of the data in that collection. N2T.net and Identifiers.org recently joined forces [38] to harmonize identifiers in the same way by using the same prefixes. As part of this partnership, they have both have adopted simple syntax that gives users finer grained control, to request to be directed to a specific source of the data; for instance, specifying the primary source of the data whether or not it has the best record of up-time.

Redistributors of data should monitor their references to other sources; any “dead” links should be reported to the original data provider. If the original provider does not fix the broken link, your reference to it should be marked obsolete both visibly (for user interaction and/or interpretation), and within any accompanying metadata (for computational interaction and/or propagation). Differentiate the identifiers that are internally linked within your application from those identifiers linked outside your application. One way to do this is by using the linkout icon; consider opening all external links in a new browser window or tab in order to avoid confusion.

## Conclusion

Better identifier design, provisioning, documentation, and referencing can address many of the identifier problems encountered in the life science data cycle—leading to more efficient and effective science. However, best practices will not be adopted on the basis of their community benefit alone; the practices must be both easy and rewarding to the groups that do the implementing. In the broader context of scholarly publishing, this is just what DOIs afford; DOIs succeeded because they were well aligned with journals’ business goals (tracking citations) and because the cost was worth it to them. However, in the current world where everyone is a data provider, alignment with business goals is still being explored: meta resolvers can provide a use case for journals and websites seeking easier access to content, while software applications leverage these identifier links to mine for knowledge.

We recognize that improvements to the quality, diversity, and uptake of identifier tooling would lower barriers to adoption of the lessons presented here (S4 Text). Those that issue data identifiers face different challenges than do those referencing data identifiers. We understand there are ecosystem-wide challenges and we will undertake to address these gaps in the relevant initiatives (S1 Text). We also recognize the need for formal software-engineering specifications of identifier formats and/or alignment between existing specifications. Here, we implore all participants in the scholarly ecosystem—authors, data creators, data integrators, publishers, software developers, resolvers—to aid in the dream of identifier harmony and hope that this paper can catalyze such efforts.

## Supporting information

S1 Table Glossary of web technology terms. Technical terms relevant to web technology and identifiers (those such as Local ID that appear in fixed-width font throughout the paper) are formally defined herein.
(PDF)

S2 Table Prefix and URI pattern registries in life science. (A) Primary registries for prefixes and/or URI patterns and (B) secondary sources of same.
(PDF)

S3 Table Questions that good identifier documentation should answer. Despite its usefulness to data integrators, identifier documentation is often overlooked and underspecified. This table describes the questions that comprehensive identifier documentation should answer.
(PDF)

S1 Text Initiatives relevant to identifiers. International initiatives that are working to address various issues related to identifiers in the scholarly landscape.
(PDF)

S2 Text Utility of CURIEs. The features that make for a good persistent URI also make for good CURIEs: desirable features include lack of semantics in both the URI pattern and the local ID (Lesson 4), absence of characters after the local ID (Lesson 5), omission of problematic characters, etc. (Lesson 5). CURIEs can complement http URIs in important ways for curators and data integrators: location-independence, brevity, clues to collapse equivalents. These features are described.
(PDF)

S3 Text Things to consider when choosing a resolver approach. There are basically three kinds of approaches to serving URIs on the web: (A) “native” URIs that require no redirection at all (as in Fig 4, ZFIN), (B) “in house” URIs that redirect internally (as in Fig 4, Ensembl), and (C) schemes using an external resolving authority (as in Fig 4, Biosamples). A list of considerations for which approach and which services to adopt.
(PDF)

S4 Text Current and future efforts that would help lower barriers to adoption. A list of current and future tooling and technologies that could help lower barriers to the adoption of identifier best practice.
(PDF)

## Abbreviations

ABC: address bar copy
API: application programming interface
ASCII: American Standard Code for Information Interchange
CURIE: Compact Uniform Resource Identifier
DOI: Digital Object Identifier
FAIR: findability, accessibility, interoperability, and reuse
HTTP: hypertext transfer protocol
IETF: Internet Engineering Task Force
JSON-LD: JavaScript Object Notation for Linked Data
LINCS: Library of Integrated Network-based Cellular Signatures
local ID: local identifier
MGI: Mouse Genome Informatics
PDF: portable document format
REST: representational state transfer
URI: nique Resource Identifier
URL: Uniform Resource Locator
W3C: world wide web consortium
ZFIN: Zebrafish Identification Network
