# Supplementary material for: Identifiers for the 21st century: How to design, provision, and reuse persistent identifiers to maximize utility and impact of life science data
Source: PLoS Biol. 2017 Jun 29;15(6):e2001414. doi: 10.1371/journal.pbio.2001414 (PMC5490878; doi:10.1371/journal.pbio.2001414)
Supplement: S1 Table — Technical terms relevant to web technology and identifiers (those such as Local ID that appear in fixed-width font throughout the paper) are formally defined herein. (PDF) [file pbio.2001414.s001.pdf]

## S1 Table. Glossary of web technology terms

| Term | Definition |
|---|---|
| `Access URL` | The URL of the page to which the http URI is ultimately redirected. Such a page is often referred to as a `landing page` (see below). While experts may differ about what is and is not a landing page, the most important definition. |
| `Alternate identifier` | A 3rd-party-issued identifier that refers to an entity that already has its own (`indigenous`) identifier. See also `Surrogate identifier`. |
| `ASCII` | ASCII is a 8-bit character encoding, the first 7-bits define a stable set containing 128 characters[1]. It contains the numbers from 0-9, the uppercase and lowercase English letters from A to Z, and some special characters. UTF-8 keeps the first 7-bits of ASCII as is and includes non-ASCII characters that may be used in Internationalized Resource Identifiers (IRI), however since non-ASCII characters are not allowed in URIs, ASCII is the least problematic choice. |
| `Base identifier (aka base record)` | An identifier that intentionally has no version information embedded. For databases that have an entity-level versioning policy, the "base identifier" would have no versioning embedded local part (through dot suffixing); eg, in UniProt, a base identifier would be http://www.uniprot.org/uniprot/P12345 and a corresponding versioned identifier http://www.uniprot.org/uniprot/P12345.1. For databases that have release-level versioning, the base resource http://ensembl.org/id/ENSMUSG00000033577 would be http://e85.ensembl.org/id/ENSMUSG00000033577. The base identifier serves two purposes. 1) redirection to the most current version, and 2) convenience of omitting version if that level of detail is not important for a particular use case. Base identifiers are useful even in cases where versioning is done solely in metadata. |
| `content drift` | "The resource identified by a URI may change over time and hence, the content at the end of the URI may evolve, even to such an extent that it ceases to be representative of the content that was originally referenced."[2] |
| `Content negotiation` | "Content negotiation is a mechanism defined in the HTTP specification that makes it possible to serve different versions of a document (or more generally, a resource representation) at the same URI, so that user agents can specify which version fit their capabilities the best."[3] [4] |
| `Cross reference` | A reference to an `entity` in a 3rd party `database, repository, registry, or ontology`; classical cross references are not accompanied by recapitulated data from the native `entity`. |
| `CURIE prefix (see also prefixed URI)` | • deterministically expandable to a URI pattern (see below) which is the basis for the CURIE's global uniqueness<br>• a mnemonic that helps in human communication<br>• documented and aspirationally globally unique<br>• documented in terms of its case convention<br>• conforms to the rules of an XML QName (e.g. does not contain ':') |
| `Domain Name System (DNS)` | The Domain Name System (DNS) is a hierarchical distributed naming system for computers, services, or any resource connected to the Internet or a private network. It associates various information with domain names assigned to each of the participating entities.[5] |
| `Entity` | An identifiable unit, for instance in a database, registry, repository, or ontology. Entities can be of different types: Digital entities include files, images, video, etc. Physical entities include things like preserved specimens, individual living specimens, and strains or lines of living specimens. |
| `HTTP Status codes` | When a web resource is requested, the response falls into one of five high-level categories, or "HTTP status codes": 1) informational, 2) success, 3) redirection, 4) client error, 5) server error. For instance, a '302 redirect' means that the resource has moved temporarily; '301 redirect' means the move is permanent. This distinction enables search engines to keep the old page, or replace it with the one at the new location.[6] |

| | |
|---|---|
| **JSON-LD** | JSON-LD, or JavaScript Object Notation for Linked Data, is a method of encoding Linked Data using JSON. It was a goal to require as little effort as possible from developers to transform their existing JSON to JSON-LD. This allows data to be serialized in a way that is similar to traditional JSON. [7] |
| **label** | A human-readable version of a resource's name. Labels should be displayed where human comprehension is important, but labels should be backed by identifiers. In the context of the Semantic Web, labels are often instances of rdf:Property. |
| **landing page** | The JDDCP recommends that citations be human *and* machine readable. It's very hard to ensure that all machines (or people) are ready to consume, interpret or access the data. A landing page provides any additional information that is required for these points. A landing page also can serve as the intermediary for complex data packages, e.g., .zip, .tar, gz, to provide a unique point of access. Landing pages should ensure that both the metadata and the data are "Machine accessible", i.e., that the landing page provides access by well-documented Web services to data and metadata stored in a robust repository, independent of browser access by humans. Specific recommendations for how to achieve these goals may be found in Starr et al (2015)[8]. The DCIP Expert Group on Repository Metadata will also be issuing a set of guidelines in the near future. The URL that corresponds to the landing page is an "`access URL`". |
| **Local Identifier (Local ID)** | An identifier that is only guaranteed to be unique within a single database. (See Box 1 and Figure 1). While the concept has historical precedent, we are introducing the term itself for the first time here. In prior versions of this paper, we referred to it as LRI (Local Resource Identifier). |
| **link rot** | "Link rot (or linkrot), also known as link death, link breaking or reference rot, refers to the process by which hyperlinks on individual websites or the Internet in general point to web pages, servers or other resources that have become permanently unavailable."[9] |
| **Persistent identifier** | The term '**persistent identifier**' is usually used in the context of digital objects that are accessible over the Internet. Typically, such an identifier is not only persistent but also actionable[10]: it is a **Uniform Resource Identifier (URI)**[11], almost alwaysusually of type http/s, that you can paste in a web browser address bar and be taken to the identified source. |
| **Permalink** | A permalink or permanent link is a URL that is intended to remain unchanged for many years into the future, yielding a hyperlink that is less susceptible to link rot. Permalinks are often rendered simply, that is, as friendly URLs, so as to be easy for people to type and remember. Most modern blogging and content-syndication software systems support such links. Sometimes URL shortening is used to create them. A permalink is a type of `persistent identifier` and the word permalink is sometimes used as a synonym of persistent identifier. More often, though, permalink is applied to persistent identifiers which are generated by a content management system for pages served by that system. This usage is especially common in the blogosphere. Such links are not maintained by an outside authority, and their persistence is dependent on the durability of the content management system itself. [12] |
| **Indigenous identifier (aka native identifier)** | An identifier issued by an entity's original or authoritative source (eg. original `database, repository, or registry`), also referred to as native identifier[13]. See also `surrogate identifier` and `alternate identifier`. |
| **prefixed URI or "CURIE" (see also CURIE prefix)** | A compact URI comprised of `<Prefix>:<Local ID>` wherein **prefix** is deterministically expandable to **a URI pattern** to yield the http URI which *alone* is the basis for the CURIE's global uniqueness. An example of a CURIE is `UniProtKB:A0A022YWF9`. Occasionally, the CURIE is the ID form that is actually used locally (see MGI, figure 2) and thus functions as a Local ID. [14] In such cases, the prefix should always appear once (MGI:3840442) it should not be needlessly duplicated (MGI:MGI:3840442). |
| **Registries and Repositories** | Databases may be classified as registries, repositories, both or neither. A registry is an indexed list of entities with pointers to their external locations. A repository internally stores the actual entities and assumes primary responsibility for them. Knowledge bases synthesize information from diverse sources. In practice, most databases combine features of the these three categories and can be differently classified depending on the entity in question: for instance, BioSamples |

| | |
|---|---|
| | DB[15] is a *repository* of BioSample information but a *registry* of the experimental data associated with those samples. |
| **URI** | An identifier that is guaranteed to be both uniform and globally unique. In this paper, we define a URI as an ASCII string that uniquely identifies a Web (not localhost) resource and also resolves to (provides or redirects to) a webpage containing information about the identified entity. Such URIs are generally of the HTTP protocol but may be other (e.g. HTTPS). Although according to their original specification, URIs may either be of type URN or URL, common usage of the term **'URI'** almost always means those of type URL only. We have further distinguished between a URIs and 'access URLs', not because their anatomy or technical specification differs, but because their purpose differs. URIs may and should be used for identification purposes because they are designed to be persistent. Access URLs on the other hand are ephemeral and should therefore not be used for identification purposes. It can be difficult or impossible for a user to determine whether a given URL is an access URL or a URI. In native resolution (**ZFIN, Fig. 1a**), access URL and URI are exactly the same; this approach reduces the likelihood that an ephemeral address will be used for identification purposes. Providers that choose redirection strategies (**Fig 1b-1e**) for their URIs must be vigilant about documentation for users. [16] |
| **URI pattern** | A URI pattern (sometimes referred to as a "resolving namespace" a fixed sequence of characters that can be used to resolve a database's local IDs. In this paper, we mean "URI pattern" to mean the simplest scenario wherein the pattern can be prepended to the local ID (or to the part of the CURIE that follows the colon, if different). See **Fig. 2 for examples**. In all cases, the **URI pattern** must be exactly as it appears in the URI: it must include the protocol (e.g. http://) and, if applicable, trailing slash or other delimiters. Some providers require additional characters *after* the Local ID is appended; this should be strongly avoided as it requires the URI patterns to contain tokens that are replaced eg. example.org/$id/view; token replacement works fine in custom code but is not supported in normal contexts such as JSON-LD, XML etc. The combination of documented URI patterns and local ID regular expressions makes it possible for consumers/integrators to validate any referenced http URIs they happen to be using. |
| **Surrogate identifier** | A 3rd-party-issued identifier that refers to an entity that does not already have its own (native) identifier. See also Alternative identifier. Surrogate identifiers are most often issued when the identifier that is needed by third parties is more granular, or less granular than the one provided by the native source. For instance, many antibody manufacturers have an online catalogs with a single PDF containing hundreds of antibodies each with a catalog number. However, it is rare for the manufacturers to provide corresponding webpages for each product. Thus in order to ensure that the identifiers are uniquely referenceable and resolvable to a webpage, the http://antibodyregistry.org/ created surrogate URIs containing the local catalog numbers as advertised by the manufacturer[17]. This was done so that antibodies could be more reliably referenced in the literature and their usage better tracked.[18] |
| **Tombstone page** | A page which continues to resolve after the corresponding entity has been deleted. It should provide the reason that the object was deleted and some basic metadata about the object |
| **Web Resource** | "Every 'thing' or entity that can be identified, named, addressed or handled, in any way whatsoever, on the web at large, or in any networked information system." [19] |
| **XRef** | Also known as "external reference" or "cross reference", XRefs are references from one database to a record in another database. |


1. Wikipedia contributors. ASCII. In: Wikipedia, The Free Encyclopedia [Internet]. 3 Mar 2017 [cited 6 Mar 2017]. Available: http://en.wikipedia.org/w/index.php?title=ASCII&oldid=768432447

2. Klein M, Van de Sompel H, Sanderson R, Shankar H, Balakireva L, Zhou K, et al. Scholarly context not found: one in five articles suffers from reference rot. PLoS One. 2014;9: e115253.

3. Wikipedia contributors. Content negotiation. In: Wikipedia, The Free Encyclopedia [Internet]. 26 Feb 2017 [cited 6 Mar 2017]. Available: http://en.wikipedia.org/w/index.php?title=Content_negotiation&oldid=767497641

4. Berrueta D, Fernández S, Frade I. Cooking HTTP content negotiation with Vapour. of 4th Workshop on Scripting for the …. Citeseer; 2008; Available: http://citeseerx.ist.psu.edu/viewdoc/download?doi=10.1.1.142.8172&rep=rep1&type=pdf

5. Wikipedia contributors. Domain Name System. In: Wikipedia, The Free Encyclopedia [Internet]. 4 Mar 2017 [cited 6 Mar 2017]. Available: https://en.wikipedia.org/w/index.php?title=Domain_Name_System&oldid=768585053

6. Leach PJ, Berners-Lee T, Mogul JC, Masinter L, Fielding RT, Gettys J. Hypertext Transfer Protocol--HTTP/1.1. 1999; Available: https://tools.ietf.org/html/rfc2616

7. JSON-LD 1.0 [Internet]. [cited 6 Mar 2017]. Available: https://www.w3.org/TR/json-ld/

8. Starr J, Castro E, Crosas M, Dumontier M, Downs RR, Duerr R, et al. Achieving human and machine accessibility of cited data in scholarly publications. PeerJ Comput Sci. 2015;1. doi:10.7717/peerj-cs.1

9. Wikipedia contributors. Link rot. In: Wikipedia, The Free Encyclopedia [Internet]. 9 Feb 2017 [cited 6 Mar 2017]. Available: https://en.wikipedia.org/w/index.php?title=Link_rot&oldid=764507867

10. Wikipedia contributors. Persistent identifier. In: Wikipedia, The Free Encyclopedia [Internet]. 8 Oct 2016 [cited 13 Feb 2017]. Available: https://en.wikipedia.org/w/index.php?title=Persistent_identifier&oldid=743128540

11. Berners-Lee T. Uniform Resource Locators: A unifying syntax for the expression of names and addresses of objects on the network. 1993; Available: https://www.w3.org/Addressing/URL/uri-spec.html

12. Wikipedia contributors. Permalink. In: Wikipedia, The Free Encyclopedia [Internet]. 15 Sep 2016 [cited 6 Mar 2017]. Available: https://en.wikipedia.org/w/index.php?title=Permalink&oldid=739572533

13. Guralnick RP, Cellinese N, Deck J, Pyle RL, Kunze J, Penev L, et al. Community next steps for making globally unique identifiers work for biocollections data. Zookeys. 2015; 133–154.

14. Birbeck M, McCarron S. CURIE Syntax 1.0. W3C Candidate Recommendation CR-curie-20090116. 2009; Available: https://www.w3.org/TR/curie/

15. EMBL-EBI. Biosamples < EMBL-EBI [Internet]. [cited 6 Mar 2017]. Available: https://www.ebi.ac.uk/biosamples

16. Wikipedia contributors. Uniform Resource Identifier. In: Wikipedia, The Free Encyclopedia [Internet]. 6 Mar 2017 [cited 6 Mar 2017]. Available: https://en.wikipedia.org/w/index.php?title=Uniform_Resource_Identifier&oldid=768870095

17. The Antibody Registry [Internet]. [cited 6 Mar 2017]. Available: http://antibodyregistry.org/

18. Frontiers | An antibody registry for biological sciences [Internet]. [cited 17 Feb 2017]. doi:10.3389/conf.fninf.2011.08.00067

19. Wikipedia contributors. Web resource. In: Wikipedia, The Free Encyclopedia [Internet]. 15 Jan 2017 [cited 6 Mar 2017]. Available: http://en.wikipedia.org/w/index.php?title=Web_resource&oldid=760120815
