# Supplementary material for: Identifiers for the 21st century: How to design, provision, and reuse persistent identifiers to maximize utility and impact of life science data
Source: PLoS Biol. 2017 Jun 29;15(6):e2001414. doi: 10.1371/journal.pbio.2001414 (PMC5490878; doi:10.1371/journal.pbio.2001414)
Supplement: S2 Table — (A) Primary registries for prefixes and/or URI patterns and (B) secondary sources of same. (PDF) [file pbio.2001414.s002.pdf]

S2 Table. Prefix and URI pattern registries

S2a Table. Formal registries

Prefix registry	Scope	Registration URL	Note	Registers Native Prefix	Registers URI pattern	Functions as a resolver
Identifiers.org[1]	Life sciences	https://sourceforge.net/p/identifiers-org/new-collection/	Manually curated. Core OBO foundry namespaces are imported periodically.	yes	yes	yes
n2t.net[2]	Cross-domain Name-to-Thing resolver	http://n2t.net	Supports a combination of per-identifier and per-scheme (rule) redirects.	yes	yes	yes
Data Hub[3]	Cross-domain	http://datahub.io	Datasets may be uploaded or registered for free. The Data Hub registry is used to populate the linked open data cloud.[4]	yes	yes	no
OBO foundry[5]	Bio-Ontologies	http://www.obofoundry.org/join.shtml	Each ontology in OBO requires an “ID space” which is unique across all ontologies in OBO. Not all ontologies are eligible for inclusion.	yes (“ID space”)	no	yes, OBO PURL only
Bioportal[6]	Bio-Ontologies	http://bioportal.bioontology.org/login?redirect=/ontologies/new	Each ontology in BioPortal requires a “Short ID” which is unique across all ontologies in BioPortal.	yes (“Short ID”)	no	yes, Bioportal PURL only
Prefix.cc[7]	Cross-domain	http://prefix.cc/	Designed for Semantic Web practitioners. Accepts, short, memorable prefixes only, punctuation not allowed. Prefix assignments are ranked according to community voting.	yes	yes	no
Linked Open Vocabularies[8]	Cross-domain	http://lov.okfn.org/dataset/lov/	Designed for Semantic Web practitioners. Vocabularies relevant to linked data.	yes	yes	no

S2b Table. Hybrid registries

There are other databases and web applications that leverage/mirror the `prefixes/uri patterns` served from the above registries, but that do not register any new ones themselves. This hybrid approach requires post-hoc coordination. The ongoing coordination and aggregation of information from various prefixing authorities is important to further minimize collisions.

Prefix registry	Scope	Registration URL	Note	Registers Native Prefix	Registers URI pattern	Functions as a resolver
Prefix Commons Biocontext	Primarily lifesciences	https://github.com/prefixcommons/biocontext/blob/master/README.md	Enables any registry or integrator to declare the mappings they issue and happen to use			no
Ontology Lookup Service	Ontologies used by EMBL-EBI	n/a	Scope is for ontologies used in molecular biology	no	no	yes
BioSharing	Life sciences - policies, standards and databases	https://www.biosharing.org/new/	Manually curated crowd-sourcing approach. Periodically synchronized with other sources such as Identifiers.org. Each	yes	no	yes, BioSharing PURL only

			BioSharing record is registered with a short ID, which is unique across all of BioSharing.			
Gene Ontology Prefix Registry	Identifiers that use GO or that are used by GO	https://github.com/geneontology/go-site/blob/master/metadata/db-xrefs.yaml (click on the 'edit' icon)	Manually curated YAML, managed in github. Pull requests accepted. Periodically manually synchronized with other sources such as Identifiers.org	yes	yes	Yes (in a limited context, eg. Amigo)

1. Juty N, Le Novère N, Laibe C. Identifiers.org and MIRIAM Registry: community resources to provide persistent identification. Nucleic Acids Res. 2012;40: D580–6.
2. Wimalaratne S, Juty N, Kunze J, Janée G, McMurry JA, Beard N, et al. Uniform Resolution of Compact Identifiers for Biomedical Data [Internet]. bioRxiv. 2017. p. 101279. doi:10.1101/101279
3. Welcome - the Datahub [Internet]. [cited 6 Mar 2017]. Available: http://datahub.io
4. Cyganiak R, Jentzsch A. Linking open data cloud diagram. LOD Community (http://lod-cloud net/). 2011;12. Available: http://lod-cloud.net/
5. Smith B, Ashburner M, Rosse C, Bard J, Bug W, Ceusters W, et al. The OBO Foundry: coordinated evolution of ontologies to support biomedical data integration. Nat Biotechnol. 2007;25: 1251–1255.
6. Whetzel PL, Noy NF, Shah NH, Alexander PR, Nyulas C, Tudorache T, et al. BioPortal: enhanced functionality via new Web services from the National Center for Biomedical Ontology to access and use ontologies in software applications. Nucleic Acids Res. 2011;39: W541–5.
7. namespace lookup for RDF developers | prefix.cc [Internet]. [cited 6 Mar 2017]. Available: http://prefix.cc
8. Vandenbussche P-Y, Atemezing GA, Poveda-Villalón M, Vatant B. Linked Open Vocabularies (LOV): a gateway to reusable semantic vocabularies on the Web. Semantic Web. IOS Press; 2017;8: 437–452.
