# Supplementary material for: Identifiers for the 21st century: How to design, provision, and reuse persistent identifiers to maximize utility and impact of life science data
Source: PLoS Biol. 2017 Jun 29;15(6):e2001414. doi: 10.1371/journal.pbio.2001414 (PMC5490878; doi:10.1371/journal.pbio.2001414)
Supplement: S3 Table — Despite its usefulness to data integrators, identifier documentation is often overlooked and underspecified. This table describes the questions that comprehensive identifier documentation should answer. (PDF) [file pbio.2001414.s003.pdf]

S3 Table. Questions that good identifier documentation should answer

Scope	Question to answer	Recommendation
Provider	What types of entities are identified, what is the scope of these entities?*	Must include
Provider	What is your primary `URI pattern`, if only one exists? If multiple, equally-valid `URI patterns` co-exist, what are these?<br>(e.g. INSDC.org has four such schemes as the entire dataset is fully represented by each of three authorities: NCBI, ENA, and DDBJ)	Must include
Provider	Are you aware of any alternate URIs (eg. different resolvers) that other groups use for your identifiers? (Even though alternates are not recommended for use, knowing what which URIs are equivalent facilitates data integration.)	Could include
Provider	What is the `prefix` you wish others to use if they reference your entities in an abbreviated way? If this `prefix` is registered, where? What is the compact URI you wish others to use?**	Must include
Provider	What is your persistence policy regarding maintenance of the URIs? What is your persistence policy regarding the corresponding entities and metadata?	Must include
Provider	Can machine-readable representations of your entities be accessed? If so, where and in what formats?	Must include
Provider	What is the regular expression of your `Local IDs` and URIs? What do your identifiers look like. If possible provide a strict pattern to describe these identifiers.	Must include
Provider	Are there relationships between your identifiers? Where are these described?*	Should include
Provider	Under what license are identifiers made available?	Should include
Provider	Does the lifecycle of the entities potentially include versioning, splitting, merging, or deprecation? How are these changes managed, communicated, and synchronized between those using that entity?*	Must include
Provider-Redistributor	Do you identify *entities* that are also identified by others? Who are these others? Where are these mappings found and who, if anyone, maintains them?	Strongly recommended
Provider-Redistributor	Do you reference *identifiers* that are issued by other authorities? If so, in what cases? How often are the identifiers synchronized?	Must include
Provider-Redistributor	If you reference *identifiers* that are issued by other authorities, what are the mappings used for `prefix-to-URI patterns`? What is the source of these mappings (e.g. manual or identifier service). Where can your mappings be found?	Must include

* Adapted from the Linked open data institute recommendations [LODI]

**If your Local IDs already have a colon, make it clear to users what your preferred corresponding compact URI syntax is. We recommend referencing the LRI as if it were already a compact URI. For instance, the case of GO:0007049, the prefix 'GO' can be expanded to http://purl.obolibrary.org/obo/GO_ and prepended to the numeric fragment to yield http://purl.obolibrary.org/obo/GO_0007049, in accordance with their documentation.
