# Supplementary material for: Identifiers for the 21st century: How to design, provision, and reuse persistent identifiers to maximize utility and impact of life science data
Source: PLoS Biol. 2017 Jun 29;15(6):e2001414. doi: 10.1371/journal.pbio.2001414 (PMC5490878; doi:10.1371/journal.pbio.2001414)
Supplement: S1 Text — International initiatives that are working to address various issues related to identifiers in the scholarly landscape. (PDF) [file pbio.2001414.s004.pdf]

S1 Text. Initiatives relevant to identifiers

- **BD2K (Big Data 2 Knowledge)**[1]**.** This US program supports a variety of initiatives aimed at making better use of the diversity of biomedical data, including various data integration efforts.
- **BioMedBridges**[2]. This is an implementation-driven project to integrate data that facilitates translational research.
- **DataCite**[3]**:** DataCite is interested in enabling the persistent identification of data, and develops and supports the standards required to achieve this.
- **DCIP**[4]**:** The Data Citation Implementation Pilot goal is to provide basic coordination between publishers, repositories and identifier / metadata services for early adopters of data citation according to the JDDCP.
- **Diachron**[5]: DIACHRON intends to address and cope with certain issues arising from the evolution of and identification of data in a web environment.
- **ELIXIR**[6]: A pan-European research infrastructure tasked with safeguarding and managing biological data.
- **Force11**[7]: This international pan-disciplinary organization is a forum for innovations in scholarly communication, including citation of data, research resources, and other web artifacts such as software.
- **Monarch Initiative**[8]**:** A global consortium dedicated to integrating cross-species genotype-phenotype data for disease discovery.
- **RDA**[9]**:** The Research Data Alliance is a globally active alliance interested in achieving the open sharing of data across countries, technologies and research domains.
- **W3C HCLS**[10]: The World Wide Web Healthcare and Life Sciences Interest group aims to develop semantic standards for interoperability.
- **OBO Foundry**[11]**:** The OBO Foundry consortium is a collaborative of ontology developers adhering to common best practices and shared principles to ensure interoperability, including a common identifier and citation policy [12]**.**
- **GA4GH**[13]**:** The members of the Global Alliance for Genomics and Health work towards integrating and analysing genomic data.
- **JATS**[14]**:** The Journal Article Tag Suite is an application of NISO Z39.96-2015, which defines a set of XML elements and attributes for tagging journal articles and describes three article models. JATS is a continuation of the NLM Archiving and Interchange DTD work begun in 2002 by NCBI[15]. It can also be used to cite data in journals.

1. BD2K Home Page | Data Science at NIH [Internet]. [cited 6 Mar 2017]. Available: http://bd2k.nih.gov/
2. BioMedBridges [Internet]. [cited 6 Mar 2017]. Available: http://www.biomedbridges.eu/
3. DataCite Team. Welcome to DataCite [Internet]. [cited 6 Mar 2017]. Available: https://www.datacite.org
4. Data Citation Implementation Pilot (DCIP). In: FORCE11 [Internet]. 28 Sep 2015 [cited 6 Mar 2017]. Available: https://www.force11.org/group/dcip
5. DIACHRON [Internet]. [cited 6 Mar 2017]. Available: http://www.diachron-fp7.eu/
6. ELIXIR. ELIXIR Data for life [Internet]. [cited 6 Mar 2017]. Available: http://elixir-europe.org/
7. FORCE11. In: FORCE11 [Internet]. [cited 6 Mar 2017]. Available: https://www.force11.org/
8. Welcome to Monarch [Internet]. [cited 6 Mar 2017]. Available: http://monarchinitiative.org/
9. RDA | Research Data Sharing without barriers [Internet]. [cited 6 Mar 2017]. Available: https://rd-alliance.org/
10. Scott Marshall M, Stephens S. Semantic Web Health Care and Life Sciences (HCLS) Interest Group [Internet]. [cited 6 Mar 2017]. Available: http://www.w3.org/blog/hcls/

11. Wg OT. The OBO Foundry [Internet]. [cited 6 Mar 2017]. Available: http://obofoundry.org

12. Hanedel Ma Mungall C. Identifier Citation Policy [Internet]. 2015 [cited 6 Mar 2017]. Available: http://obofoundry.org/id-policy.html

13. Home | Global Alliance for Genomics and Health [Internet]. [cited 6 Mar 2017]. Available: http://genomicsandhealth.org

14. Mietchen D, McEntyre J, Beck J, Maloney C, Force11 Data Citation Implementation Group. Adapting JATS to support data citation. National Center for Biotechnology Information (US); 2015.

15. Journal Article Tag Suite [Internet]. [cited 6 Mar 2017]. Available: https://jats.nlm.nih.gov/
