# Supplementary material for: Identifiers for the 21st century: How to design, provision, and reuse persistent identifiers to maximize utility and impact of life science data
Source: PLoS Biol. 2017 Jun 29;15(6):e2001414. doi: 10.1371/journal.pbio.2001414 (PMC5490878; doi:10.1371/journal.pbio.2001414)
Supplement: S2 Text — The features that make for a good persistent URI also make for good CURIEs: desirable features include lack of semantics in both the URI pattern and the local ID (Lesson 4), absence of characters after the local ID (Lesson 5), omission of problematic characters, etc. (Lesson 5). CURIEs can complement http URIs in important ways for curators and data integrators: location-independence, brevity, clues to collapse equivalents. These features are described. (PDF) [file pbio.2001414.s005.pdf]

S2 Text. Utility of CURIEs

The features that make for a good persistent URI also make for good CURIEs: desirable features include lack of semantics in both the `URI pattern` and the `local ID` **(Lesson 4)**, absence of characters after the local ID (**Lesson 5**), omission of problematic characters etc **(Lesson 5)**. `CURIEs` can complement http URIs in important ways for curators and data integrators:

A. **Brevity.** In the life sciences, prefixed identifier forms are traditionally favored over http URIs in curation tasks; for instance, within spreadsheets, online lab notebooks, and anywhere where identification is a core concern but where screen real estate is limited.
B. **Location-independence.** Third-party data integrators often add knowledge on top of existing identifiers, for instance as MonarchInitiative.org does with OMIM. But if Monarch's URIs instead included the embedded http URI of the OMIM source dataset it would look like https://monarchinitiative.org/uri=http://omim.org/entry/154700 instead of like https://monarchinitiative.org/OMIM:154700. If the OMIM ID were not converted to its CURIE form, the resulting URI in Monarch would be a) very long b) permanently vulnerable to any volatility in the original source URI. Encoding the prefix mappings for the sources dynamically provides both simplicity and
C. **Clues for collapsing equivalents.** Due to a lack of awareness and to evolving implementations and collection scope, it is exceptionally rare that only a single http URI is used for an entity. Although it is difficult to reliably 'normalize' equivalent URIs[1] that are syntactically different, the use of CURIEs can provide clues that facilitate it.

1. Wikipedia contributors. URL normalization. In: Wikipedia, The Free Encyclopedia [Internet]. 13 Jan 2017 [cited 14 Feb 2017]. Available: https://en.wikipedia.org/w/index.php?title=URL_normalization&oldid=759921577
