# Supplementary material for: Identifiers for the 21st century: How to design, provision, and reuse persistent identifiers to maximize utility and impact of life science data
Source: PLoS Biol. 2017 Jun 29;15(6):e2001414. doi: 10.1371/journal.pbio.2001414 (PMC5490878; doi:10.1371/journal.pbio.2001414)
Supplement: S3 Text — There are basically three kinds of approaches to serving URIs on the web: (A) “native” URIs that require no redirection at all (as in Fig 4, ZFIN), (B) “in house” URIs that redirect internally (as in Fig 4, Ensembl), and (C) schemes using an external resolving authority (as in Fig 4, Biosamples). A list of considerations for which approach and which services to adopt. (PDF) [file pbio.2001414.s006.pdf]

S3 Text. Things to consider when choosing a resolver approach

There are basically three kinds of approaches to serving URIs on the web: (a) "native" URIs that require no redirection at all (as in Fig. 1, ZFIN). (b) "in house" URIs that redirect internally (as in Fig. 1, Ensembl); and (c) schemes using an external resolving authority (as in Fig. 1, Biosamples). Representative resolver authorities that meet the JDDCP (https://www.force11.org/datacitation, [1]) criteria are e.g. DOI (DataCite[2], CrossRef[3]), Identifiers.org[4], Handle.net[5], PURL[6], EPIC[7], N2T[8] and NBN[9]; these are described in Starr et al[10]. Additional resolver authorities that meet the criteria but which are not described therein are EPIC[11] and w3id[12]. Note that PURLs under the authority of PURL.org had gone into read-only mode and were therefore no longer adherent to the JDDCP principles; however, the InternetArchive[13] has assumed responsibility for them as of September 2016[14].

Below are some additional criteria you may want to consider in choosing one of these resolvers.

- Does the resolver retain the native Local Resource Identifier that you issue (eg. identifiers.org, n2t.net), or does it instead issue a new one? (eg. DOI).
    - If the resolver *does* issue a new identifier, what is the typical turnaround time between request and fulfilment? Can you obtain an identifier before you yourself need to use it?
- Would you or your institution need to pay fixed/variable costs to have your identifiers resolved? If the service is free for those that need their identifiers resolved, who pays to maintain the service?
- Is the service capable of issuing and managing identifiers in the kinds of volume you would require?
- Change management policy
    - Will you need to change the data which is referenced by the URI, and if so does the resolving system under consideration permit such change?
    - Is the object to which the URI resolves allowed to be removed?
    - Does the resolver support numerical suffixing for versions of the LRI?
    - If new LRIs are issued for each version of an entity, how can versions be related to each other?
- Will you require the resolver to support multiple resolving locations (mirrors)?
- Does the resolver support content negotiation at resolver's HTTP URI?
- Does the resolver collect, index, and/or curate metadata about individual entities?
    - If so, is the metadata that is collected relevant for the types of entities identified?
- Does the resolver collect, index, and/or curate metadata about collections of entities (e.g. whole databases)?
    - If so, is the metadata that is collected relevant for the type of collection?
- Does the resolver support controlled access for confidential data?
- Is the resolver cross-discipline?

1. Joint Declaration of Data Citation Principles - FINAL. In: FORCE11 [Internet]. 30 Oct 2013 [cited 6 Mar 2017]. Available: https://www.force11.org/datacitation

2. DataCite Team. Welcome to DataCite [Internet]. [cited 6 Mar 2017]. Available: https://www.datacite.org

3. Crossref team. Metadata enables connections - Crossref. In: www.crossref.org [Internet]. [cited 6 Mar 2017]. Available: http://www.crossref.org/

4. EMBL-EBI. Identifiers.org < EMBL-EBI [Internet]. [cited 6 Mar 2017]. Available: http://identifiers.org/

5. Handle.Net Registry [Internet]. [cited 6 Mar 2017]. Available: http://handle.net/

6. PURL Administration [Internet]. [cited 6 Mar 2017]. Available: https://archive.org/services/purl/

7. Persistent Identifiers for eResearch [Internet]. [cited 6 Mar 2017]. Available: http://www.pidconsortium.eu/

8. Name-to-Thing (N2T) Identifier Resolver Home Page [Internet]. [cited 6 Mar 2017]. Available: http://n2t.net/

9. BioModels.net team. MIRIAM Registry [Internet]. [cited 6 Mar 2017]. Available: http://identifiers.org/nbn/

10. Starr J, Castro E, Crosas M, Dumontier M, Downs RR, Duerr R, et al. Achieving human and machine accessibility of cited data in scholarly publications. PeerJ Comput Sci. 2015;1. doi:10.7717/peerj-cs.1

11. Persistent Identifiers for eResearch [Internet]. [cited 6 Mar 2017]. Available: http://www.pidconsortium.eu/

12. w3id.org - Permanent Identifiers for the Web [Internet]. [cited 6 Mar 2017]. Available: https://w3id.org/

13. Internet Archive: Digital Library of Free Books, Movies, Music & Wayback Machine [Internet]. [cited 6 Mar 2017]. Available: https://archive.org/

14. Graham M. Persistent URL Service, purl.org, Now Run by the Internet Archive | Internet Archive Blogs [Internet]. [cited 6 Mar 2017]. Available: https://blog.archive.org/2016/09/27/persistent-url-service-purl-org-now-run-by-the-internet-archive/
