# Supplementary material for: Identifiers for the 21st century: How to design, provision, and reuse persistent identifiers to maximize utility and impact of life science data
Source: PLoS Biol. 2017 Jun 29;15(6):e2001414. doi: 10.1371/journal.pbio.2001414 (PMC5490878; doi:10.1371/journal.pbio.2001414)
Supplement: S4 Text — A list of current and future tooling and technologies that could help lower barriers to the adoption of identifier best practice. (PDF) [file pbio.2001414.s007.pdf]

S4 Text. Current and future efforts that would help lower barriers to adoption

Current efforts

- **Registries, 3rd party resolvers:** A list of identifier resolvers and identifier registries is in **S3 Table**.
- **PICR** [1]**:** Protein Identifier Cross-Reference Service has a service that returns identifier mappings, optionally including deleted ones. PICR or a similar service could be developed to have broader scope.
- **HCLS** [2]**:** Health Care and Life Sciences dataset descriptions provide a standard representation of the original sources of data (and therefore identifiers) in any integrated dataset.
- **JATS** [3]**:** In the context of the literature, Journal Article Tag Suite provides a standard way for data citations to be represented in the literature, facilitating credit and reward mechanisms. However, outside of the literature, referencing and display is primarily an issue of increasing awareness.
- **BioSchemas.org**[4] is promoting more consistent adoption of schema.org markup in the life sciences. Markup can facilitate more transparent provenance and credit mechanisms of integrated data, as well as optimizing data for discovery by search engines, whether Google, or others.

Future efforts

- **Identifier validator:** Identifier designers could help data producers choose the design that best suits their particular use case, validators could determine whether an existing identifier is valid according to a published scheme.
- **Embeddable citation widgets or citation markup** could help providers display citation information, clearly and consistently.
- **Archiving services: For archival of content, client-facing services include the Memento web protocol**[5]. We authors of this paper are not aware of any existing platforms that providers can outsource their content archiving to, but such a service may be worthwhile. Another function for archival services is for maintaining a robust network of linked entities. In this case, full archival of content may not be needed. Rather, resolver and/or indexing services may provide “tombstone pages” with essential metadata so that these entities can still be resolved.

1. EBI Web Team. Protein Identifier Cross-Reference Service [Internet]. [cited 8 Feb 2017]. Available: http://www.ebi.ac.uk/Tools/picr/RESTDocumentation.do

2. W3C. Dataset Descriptions: HCLS Community Profile [Internet]. 2015. Available: https://htmlpreview.github.io/?https://github.com/indiedotkim/HCLSDatasetDescriptions/blob/master/Overview.html#s6_3

3. Mietchen D, McEntyre J, Beck J, Maloney C, Force11 Data Citation Implementation Group. Adapting JATS to support data citation. National Center for Biotechnology Information (US); 2015.

4. BioSchemas [Internet]. [cited 8 Feb 2017]. Available: http://bioschemas.org/

5. Sanderson R, Phillips M, Van de Sompel H. Analyzing the Persistence of Referenced Web Resources with Memento [Internet]. arXiv [cs.DL]. 2011. Available: http://arxiv.org/abs/1105.3459
